# Barcode demultiplexing of nanopore sequencing raw signals by unsupervised machine learning

**DOI:** 10.3389/fbinf.2023.1067113

**Published:** 2023-04-27

**Authors:** Daniele M. Papetti, Simone Spolaor, Iman Nazari, Andrea Tirelli, Tommaso Leonardi, Chiara Caprioli, Daniela Besozzi, Thalia Vlachou, Pier Giuseppe Pelicci, Paolo Cazzaniga, Marco S. Nobile

**Affiliations:** ^1^ Department of Informatics, Systems, and Communication, University of Milano-Bicocca, Milan, Italy; ^2^ Microsystems, Eindhoven University of Technology, Eindhoven, Netherlands; ^3^ Institute for Complex Molecular Systems (ICMS), Eindhoven University of Technology, Eindhoven, Netherlands; ^4^ Department of Experimental Oncology, IEO European Institute of Oncology IRCCS, Milan, Italy; ^5^ European School of Molecular Medicine (SEMM), Milan, Italy; ^6^ International School for Advanced Studies (SISSA), Trieste, Italy; ^7^ Center for Genomic Science of IIT@SEMM, Istituto Italiano di Tecnologia (IIT), Milan, Italy; ^8^ Department of Oncology and Hemato-Oncology, University of Milan, Milan, Italy; ^9^ Bicocca Bioinformatics, Biostatistics and Bioimaging (B4) Research Center, Milan, Italy; ^10^ Department of Human and Social Sciences, University of Bergamo, Bergamo, Italy; ^11^ Department of Environmental Sciences, Informatics, and Statistics, Ca’ Foscari University of Venice, Venice, Italy; ^12^ Department of Industrial Engineering and Innovation Sciences, Eindhoven of University of Technology, Eindhoven, Netherlands

**Keywords:** nanopore, unsupervised learning, autoencoder, self-organising map, complexity reduction, RNA barcoding, scRNA-seq, artificial intelligence

## Abstract

**Introduction:** Oxford Nanopore Technologies (ONT) is a third generation sequencing approach that allows the analysis of individual, full-length nucleic acids. ONT records the alterations of an ionic current flowing across a nano-scaled pore while a DNA or RNA strand is threading through the pore. Basecalling methods are then leveraged to translate the recorded signal back to the nucleic acid sequence. However, basecall generally introduces errors that hinder the process of barcode demultiplexing, a pivotal task in single-cell RNA sequencing that allows for separating the sequenced transcripts on the basis of their cell of origin.

**Methods:** To solve this issue, we present a novel framework, called UNPLEX, designed to tackle the barcode demultiplexing problem by operating directly on the recorded signals. UNPLEX combines two unsupervised machine learning methods: autoencoders and self-organizing maps (SOM). The autoencoders extract compact, latent representations of the recorded signals that are then clustered by the SOM.

**Results and Discussion:** Our results, obtained on two datasets composed of *in silico* generated ONT-like signals, show that UNPLEX represents a promising starting point for the development of effective tools to cluster the signals corresponding to the same cell.

## 1 Introduction

Single-cell RNA sequencing (scRNA-seq) is a widely adopted approach to carry out high-precision analysis of complex biological systems, with particular impact on stem cell biology and cancer research (Navin, 2014). In fact, the ability to measure and model gene expression profiles from individual cells allows for deconvolving heterogeneous cell types and functional populations (Paul et al., 2015; van Galen et al., 2019), especially via high-throughput, short-read approaches (e.g., Illumina Next-Generation Sequencing technology) that can process up to thousands of cells in a single experiment (Macosko et al., 2015; Zheng et al., 2017; Ziegenhain et al., 2017). In addition to transcriptomics, emerging technologies aim to integrate different types of molecular information from the same cell including, e.g., genomics, epigenomics, and proteomics profiles; such approaches hold the potential to uncover novel insights in biological systems, and represent a great promise in the field of precision medicine (Ogbeide et al., 2022).

In this context, nanopore sequencing is a recent nucleic acid sequencing approach developed by Oxford Nanopore Technologies (ONT) that allows for sequencing individual, full-length DNA or RNA molecules. The principle of this technology is based on ratcheting a nucleic acid strand through a proteic nanopore in the presence of an ionic current across the pore itself. As the molecule is threaded through the pore, the chemical composition of the nucleotides residing within the pore at any given moment alters the ionic flow. Thanks to a current sensor coupled to each pore, these current alterations are continuously recorded, producing a trace of current (picoAmperes) over time. This signal can then be translated into a nucleic acid sequence in a process called *basecalling* using dedicated algorithms. Both ONT and the scientific community have developed several alternative basecallers in an attempt to maximize the accuracy of the generated sequences.

**FIGURE 1:**
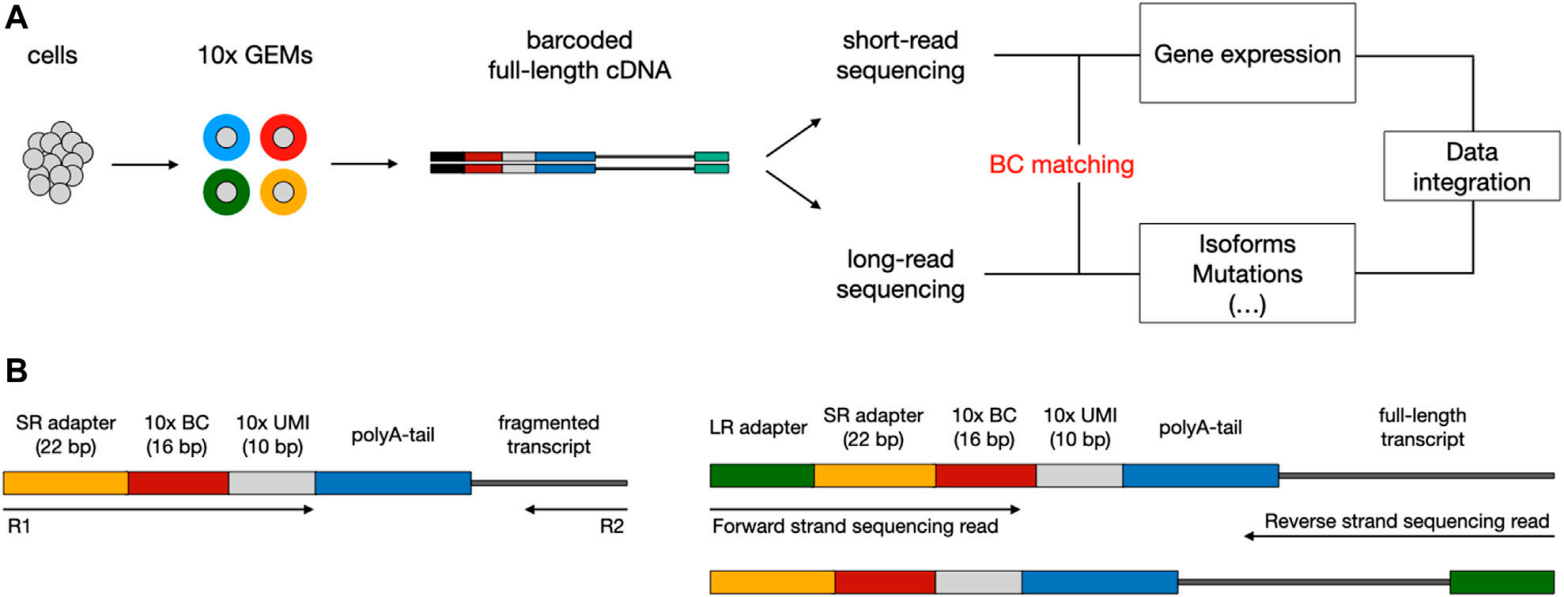
Typical workflow to couple short-read with long-read scRNA-seq using the 10x Chromium platform. **(A)** Overview of library preparation, sequencing, and downstream analysis. Single cells are captured into Gel Beads-in-emulsion (GEMs) through a microfluidic chip. Inside the GEMs, poly-adenylated mRNA transcripts undergo tagging with a cellular barcode (BC) and unique molecular identifier (UMI), followed by reverse transcription for full-length cDNA production. After the GEMs are broken, cDNA can be split between short-read (SR) and long-read (LR) sequencing. During downstream analysis, shared BCs are used to link SR and LR data, enabling the integration of multiomics information. **(B)** Structure of read templates. The SR template (left) consists of poly-adenylated fragmented transcripts attached to an Illumina adapter, BC, and UMI of fixed length, in a fixed sequence. The LR template (right) is essentially the same as the SR template but for the addition of the LR sequencing adapter and length of the transcript.

The existing sequencing platforms are characterized by different read lengths, and related benefits and flaws. *Short-read* scRNA-seq methods (i.e., Illumina) allow for effectively reconstructing the biological complexity in terms of gene expression; though, the fact they rely on sequencing just the 3′ or 5’ end of transcripts prevents an accurate exploration of other levels of heterogeneity, such as expressed mutations and alternative splicing events. On the contrary, *long-read* sequencing methods (i.e., ONT or PacBio) enable to cover the entire length of the transcripts and to perform the aforementioned analyses, but they can only exploit bulk RNA that is not multiplexed at the cellular level (Kovaka et al., 2019; Tang et al., 2020). To overcome the limits of transcript end-biased protocols, in recent years a number of experimental approaches have been devised to couple high-throughput short-read scRNA-seq with long-read sequencing, taking advantage of key features from both technologies (Gupta et al., 2018; Singh et al., 2019; Tian et al., 2021). A typical workflow consists in exploiting existing scRNA-seq platforms (mostly 10X Genomics Chromium (Zheng et al., 2017)) to produce a pool of full-length cDNA molecules, each tagged with a cellular barcode (BC), which is needed to associate each transcript with a single cell, and a unique molecular identifier (UMI), used to correct for amplification artifacts (Figure 1A). Subsequently, the barcoded cDNA can be split between short and long-read sequencing. The first step of downstream analysis aims to retrieve cellular BCs that are shared between short and long-read datasets; this is facilitated by the fact that, based on the known structure of the library, the cellular BC is expected to be in a fixed position in both short and long reads (Figure 1B). Finally, the analysis of cells that are present in both datasets allows the integration of transcriptional profiles (from short reads) with complementary layers of information (accessible from long reads).

One critical issue of this process is BCs demultiplexing, which consists of re-assigning every read to its origin sample after all reads have been sequenced together. This task is generally performed by the software of the sequencing platform, exploiting as information the sequence of the BC associated with each sample. BCs demultiplexing on Illumina data relies on the low error rate (around 0.1%) of the sequencing platform (Goodwin et al., 2016); instead, ONT data are typically noisy, because the electric signal that is generated during the sequencing needs to be deconvolved through the official ONT basecaller Guppy, which has relatively low single molecule accuracy (Kono and Arakawa, 2019). Therefore, errors are frequently introduced in the sequence of cellular BCs, which makes BC matching between Illumina and ONT data computationally challenging. To deal with this specific problem, methods for BC demultiplexing that exploit both Illumina-generated short reads and ONT-generated long reads have been recently developed, including SiCeLoRe (Lebrigand et al., 2020), FLAMES (Tian et al., 2021) and scTagger (Ebrahimi et al., 2022). However, despite differences in computational strategies and implementations, all of these methods still rely on the analysis of basecalled ONT reads, which does not eliminate the issue of inherent low accuracy, and require high-throughput short-read data to establish a trustworthy consensus.

**FIGURE 2:**
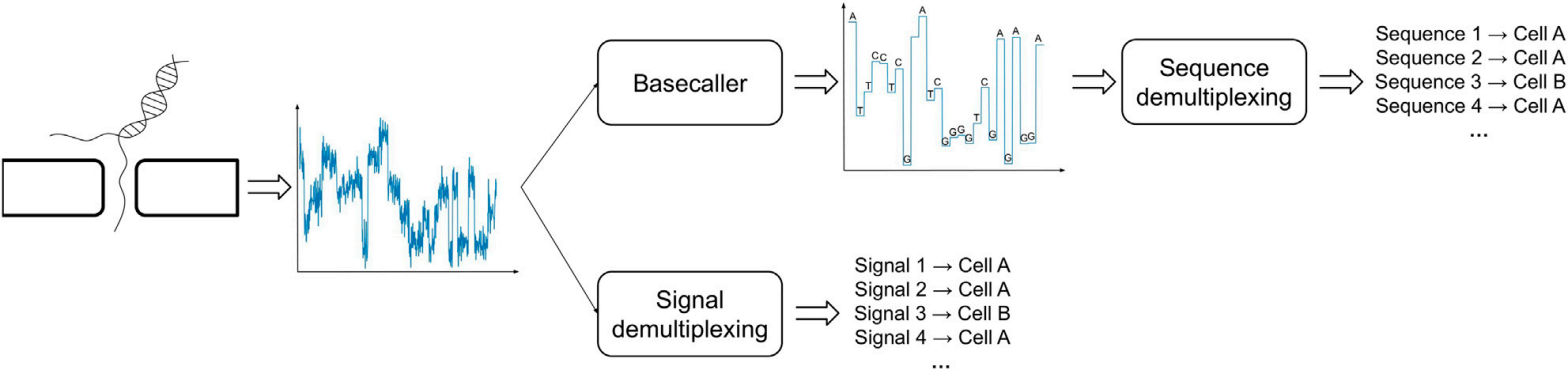
Demultiplexing of BC signals in ONT sequencing can be carried out using basecalled (top) or raw (bottom) signals.

One possible way to bypass the challenges posed by the analysis of basecalled sequences is to directly study the raw electric signals produced by the ONT sequencing (Figure 2). In order to proceed along this path one has to handle technical issues that are inherently related to the sequencing process and the type of data that is produced: the most significant of such problems is due to the fact that the speed at which a polymer transverses a pore on the capture region is not constant and is affected by a number of factors, such as the density of the electrolyte solution. A consequence of this phenomenon is the fact that the same polymer can be represented by multiple electric current signals. Therefore, in order to cluster together signals representing the same cellular BC, one has to take into account the presence of time-warping between such time-series, *i.e.*, dilation or compression of the signal along the time axis. To the best of our knowledge, DeepBinner (Wick et al., 2018) is the only published method that can directly demultiplex raw electric signals, exploiting a supervised machine learning approach based on a convolutional neural network trained on a set of very specific BCs. However, it is important to note that when ONT sequencing is not paired with a parallel Illumina sequencing run (or any other external control experiment), the BC demultiplexing problem is of inherently unsupervised nature, that is, there are no *reference* electric signals for the cellular BCs to make any meaningful comparison and the only available information is the expected number of unique Moreover, no datasets containing *labeled electric signals* are available in the literature, which are necessary to train or test any supervised machine learning approach based on the analysis of ONT-generated and not pre-processed raw signals.

In this work, we argue that the direct analysis of the raw electric signal that is produced by ONT sequencing—still immune to any basecalling error rate—might provide a means to achieve more reliable demultiplexing of BC signals generated by scRNA-seq experiments. As no tools are currently available to deal with this type of data, we developed UNPLEX, a novel method based on the combination of two unsupervised machine learning algorithms: autoencoders and self-organizing maps (SOM). Specifically, we use autoencoders to build a compact, latent representation of the BC regions of the nanopore electrical signals; then, we cluster such representations using a SOM. To test and evaluate the performance of UNPLEX we used *in silico* generated ONT-like raw signals. Our results show that this approach represents a promising starting point for the development of tools to effectively separate signals coming from the same cell, with small errors in the case of similar BC signals. Indeed, the ONT is currently evolving as the nanopore models as well as the strategies to control the speed of the DNA/RNA strand in the pore are improving.

The paper is structured as follows. In Section 2 we describe how raw signals were generated *in silico*, and explain the unsupervised machine learning approaches—autoencoders and self-organizing maps—used in this work. Then, we introduce the UNPLEX framework and describe the hyper-parameter optimization process employed to enhance UNPLEX performance. In Section 3 we show the results obtained by running UNPLEX on two *in silico* datasets. Finally, in Section 4 we discuss the results obtained and draw future research directions for this work.

## 2 Materials and methods

### 2.1 *In silico* generation of raw signals

**FIGURE 3:**
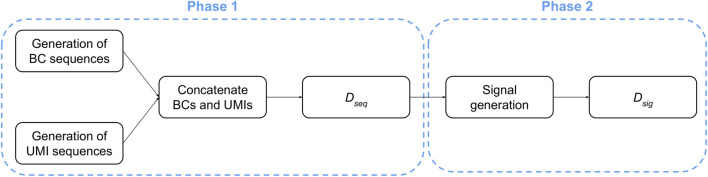
Workflow used for *in silico* generation of ONT-like signals.

The generation of raw signals consists of two consecutive phases, starting with the creation of random nucleic acids sequences, followed by the generation of the corresponding current signals (Figure 3).

Phase 1. The creation of the dataset containing the nucleic acids sequences (denoted as *D*_*seq*_ in the following) consists of three steps: (*i*) a set of *A* different BCs are created by sampling each nucleotide from a uniform distribution so that, for each position of every BC sequence, all nucleotides have the same probability to be sampled; (*ii*) a set of *B* different UMI sequences are created by sampling each nucleotide from a uniform distribution so that, for each position of every UMI sequence, all nucleotides have the same probability to be sampled; (*iii*) each UMI is concatenated to each BC.

At the end of this process, the dataset *D*_*seq*_ contains a number of sequences equal to *A* ⋅ *B*. We highlight that only the portions regarding the BC and UMI sequences were generated because they can be isolated from a ONT signal: indeed, the polyA-tail can be identified thanks to its characteristic signal, while the signal derived from the adapter is shared among all the sequences. On the contrary, it is not possible to determine where the BC signal ends and the UMI signal starts; thus, a heuristic is required to deal with signals generated from BC plus UMI sequences.

Phase 2. Starting from each sequence in *D*_*seq*_, we obtain a dataset *D*_*sig*_ of ONT-like signals by means of an iterative process that emulates the molecule threading through the pore. Namely, for each 6-*mer* of a sequence considered in the reading order, a portion of the raw signal is sampled according to the data made publicly available by Oxford Nanopore Technologies (Nanoporetech, 2017).

It is worth noting that the ONT-like signals in *D*_*sig*_ generally have different lengths because the required time for a 6-*mer* to thread through the pore varies according to many biochemical factors, e.g., the nucleic acids appearing in the 6-*mer* itself. Non-etheless, since the BC sequences and the UMI sequences have similar lengths in terms of the number of nucleic acids, we assume that also the raw signals derived from them share a similar—although not necessarily identical—length.

### 2.2 Autoencoders

Autoencoders are neural networks whose objective is to learn how to reproduce the input data (Bourlard and Kamp, 1988). Autoencoders leverage two sub-networks: the first, named encoder, receives as input the vector of original data and outputs a vector of real values that is typically smaller than the input vector; the second, named decoder, reconstructs the original data starting from the real values vector computed by the encoder. Thanks to this topology, autoencoders learn how to represent the data in a different feature space by extracting the most relevant latent features of the input data. The extracted features generally represent hidden structures within the input and allow for learning an effective encoding to regenerate the original data. Both the encoder and the decoder are simultaneously trained following the standard supervised training process of neural networks. Though, autoencoders are usually considered as an unsupervised machine learning approach since they are trained with unlabeled data. In this work, we use a convolutional autoencoder whose topology (see Supplementary Table S1) is derived from a convolutional autoencoder previously used to perform feature extraction of electroencephalography signals (Wen and Zhang, 2018). Due to the different dimensionality of the signals considered in this work and the necessity to have a succinct representation for the successive computational steps of UNPLEX, we modify the topology proposed in (Wen and Zhang, 2018) by adding a convolutional layer of 16 filters at the end of the encoder and a maximum pooling operator. This convolutional layer is used to reduce the number of filters from 64 to 16, while the maximum pooling operator halves the size of each filter of the embedding; such a topology results in a great reduction of the size of the embedding. Symmetrically, an up sampling operator and a convolutional layer are added to the decoder.

Being based on convolutional layers, the input data of the autoencoder must be of a fixed length. Since the ONT-like signals have different lengths, as described in Section 2.1, we perform a pre-processing step to produce a new dataset *D*_*tsig*_, which will contain signals whose lengths are all equal to the half length (denoted by *l*) of the longest signal in the dataset *D*_*sig*_. To this aim, we first isolate the first half of each signal: this is a heuristic used to remove a portion of the UMI signal, since the BC and UMI sequences have similar lengths. Then, each half of the signal is concatenated to itself until the signal length is equal to *l*. In case the concatenation step produces signals with a length higher than *l*, the signal is truncated at length *l*. Finally, the intensity values of the signals are normalized in the unit interval by using as the minimum (maximum) value the lowest (highest) intensity value among all signals used to train the autoencoder. It is worth noting that the isolation of the first half is performed on the signals and not on the original sequences because, in real-world applications, the information regarding the original sequences is not available.

The autoencoder is used to generate a new dataset *D*_*emb*_ by extracting the latent representations, or embeddings, of all signals belonging to *D*_*tsig*_. The dataset *D*_*emb*_ will thus contain vectors of real numbers used to train the Self-Organizing Map, as described in Section 2.3. To produce the embeddings, the dataset *D*_*tsig*_ is partitioned into a training set and validation set according to a 80 − 20 split. We exploit the Adam optimizer, and an early stopping criterion monitoring the Mean Squared Error (MSE) computed on the validation set.

### 2.3 Self-organizing maps and clustering

Self-Organizing Maps (SOMs) are a class of unsupervised neural networks that exploit a competitive learning approach to cluster the input data in a regular grid of elements called neurons (Kohonen, 1982; Kaski, 1997). Neurons correspond to real-valued vectors, whose components are usually referred to as weights. The training of a SOM is an iterative process in which the neurons are updated to better represent all the elements in a dataset that, at each iteration, are fed to the SOM to determine the so called Best Matching Unit (BMU). The BMU is the neuron that minimizes a given distance function calculated with respect to the input vector. Then, both the BMU and the neurons in its neighborhood are updated to better resemble the input vector. At the end of the training process, the neurons representing similar samples are topologically near to each other, while the neurons representing different samples are in further regions of the grid.

The performance of a SOM is influenced by many hyper-parameters, such as the number and initial value of neurons, the learning rate, the network topology, the neighborhood function, and the distance function. The weights initialization strategy is based on principal component analysis (PCA) (Jolliffe, 2002), which was proved to allow SOMs to converge starting from any initial state (Kohonen, 2013). The PCA-based strategy computes the first two eigen-vectors of *D*_*emb*_, and the weights are then sampled from the hyper-plane defined by the two eigen-vectors. The learning rate is a real-valued number that determines the magnitude of each neuron’s update and prevents the neurons from “forgetting too quickly” input vectors previously received. The topology determines how the neurons are organized in a regular grid; in this work, we consider squared tiles and hexagon-shaped tiles. The topology directly impacts on the choice of the most suitable neighborhood function, which determines how the neurons in the BMU’s neighborhood interact, compete and learn to better match the samples. The neighborhood functions are time-dependent, meaning that the size of the neighborhood they identify decreases as the training of the SOM proceeds. The neighborhood function also influences the magnitude of the update of the weights: the further from the BMU the neuron is, the weaker the update. In this work, we test four neighborhood functions: Gaussian, Bubble, Mexican hat, and Triangle (see Supplementary Table S2). The Gaussian function leverages a normal distribution to determine the intensity of the weights update: the neurons closer to the BMU (mean) are more influenced than the further neurons, while the size of the neighborhood is determined by the standard deviation. The main drawback of this function is the computational cost required to compute the exponential function of the normal distribution; therefore, for the sake of computational efficiency, we also consider the other three neighborhood functions that are approximations of the Gaussian function. Finally, we test the impact of four distance functions: Cosine, Manhattan, Euclidean, and Chebyshev (see Supplementary Table S3).

At the end of the training process, the SOM is employed to determine the BMU of each element of the input dataset, thus grouping the elements according to their representative neuron. In particular, it is possible to apply a clustering algorithm to the SOM’s neurons to obtain a mapping from the input vector to the BMU. To evaluate the quality of a clustering, extrinsic metrics can be leveraged when the ground truth for each sample is available. In this work, we use two extrinsic metrics: Adjusted Random Score (ARS) (Hubert and Arabie, 1985) and Fowlkes-Mallows Score (FMS) (Fowlkes and Mallows, 1983). ARS is an overall measure that aims to reward the clustering when: (*i*) a couple of samples is supposed to belong to the same cluster and it is correctly grouped into the same cluster; (*ii*) a couple of samples is not supposed to belong to the same cluster and it does not belong to the same cluster. Similarly, FMS considers all the possible couples of samples and rewards the clustering when the couples are correctly clustered together. Conversely, a penalization is used in FMS when two samples that should belong to different clusters are grouped together and when the samples that should belong to different clusters are grouped together.

### 2.4 Hyper-parameters optimization

The performance of any machine learning algorithm is influenced by the values of its hyper-parameters, whose tuning is typically based on a trial-and-error approach that is time-consuming, error-prone, and hampers the repeatability of the experiments (Hutter et al., 2019). The trial-and-error approach might also prevent the user to find optimal parameterizations since the search is solely guided by the user experience and insights. For these reasons, over the last decade the problem of tuning the hyper-parameters has been formulated as an optimization problem—Hyper-Parameter Optimization (HPO)—whose objective is to find a parameterization that maximizes the performance obtained by the machine learning model. The strategies proposed to solve the HPO problem range from the application of genetic programming or genetic algorithms to sequential approaches (Yu and Zhu, 2020; Alibrahim and Ludwig, 2021). In general, they evaluate and evolve a number of different parameterizations, and let the obtained performance guide the search toward more promising parameterizations. The main drawback concerns the computational cost required by this process, because for each candidate parameterization the model has to be trained and evaluated. A strategy to limit such issue is the use of surrogate models—built starting from the performance of the model obtained on a small set of parameterizations—that are cheaper to evaluate in terms of running time (Hutter et al., 2019). Though, the quality of the surrogate model is related to the number of evaluated parameterizations: the higher the number of evaluated parameterizations, the better the surrogate model.

In this work, we use the Sequential Model Based Optimization (SMBO), a general-purpose method specifically defined to optimize the hyper-parameters of an algorithm by means of a Bayesian optimization process (Hutter et al., 2011). The SMBO method can be summarized in the following steps: (*i*) build a surrogate model on the basis of the results of randomly sampled parameterizations; (*ii*) find the optimum of the surrogate model; (*iii*) evaluate the corresponding parameterization; (*iv*) update the surrogate model. Steps (*ii*)-(*iv*) are repeated until a termination criterion is met (e.g., a number of Model Evaluations (MEs) have been performed). The main advantage of this sequential approach is that the first surrogate model is built by leveraging a fraction of the MEs available. So doing, the remaining MEs are used to simultaneously update the surrogate model and effectively search for an optimal parameterization. The sequential approach also allows for performing a reduced number of MEs, which is ideal when the training and evaluation of a model are expensive in terms of computational resources. In fact, the execution of a sequential search can be interrupted at any iteration and the method provides a sub-optimal solution.

### 2.5 The UNPLEX framework

**FIGURE 4:**
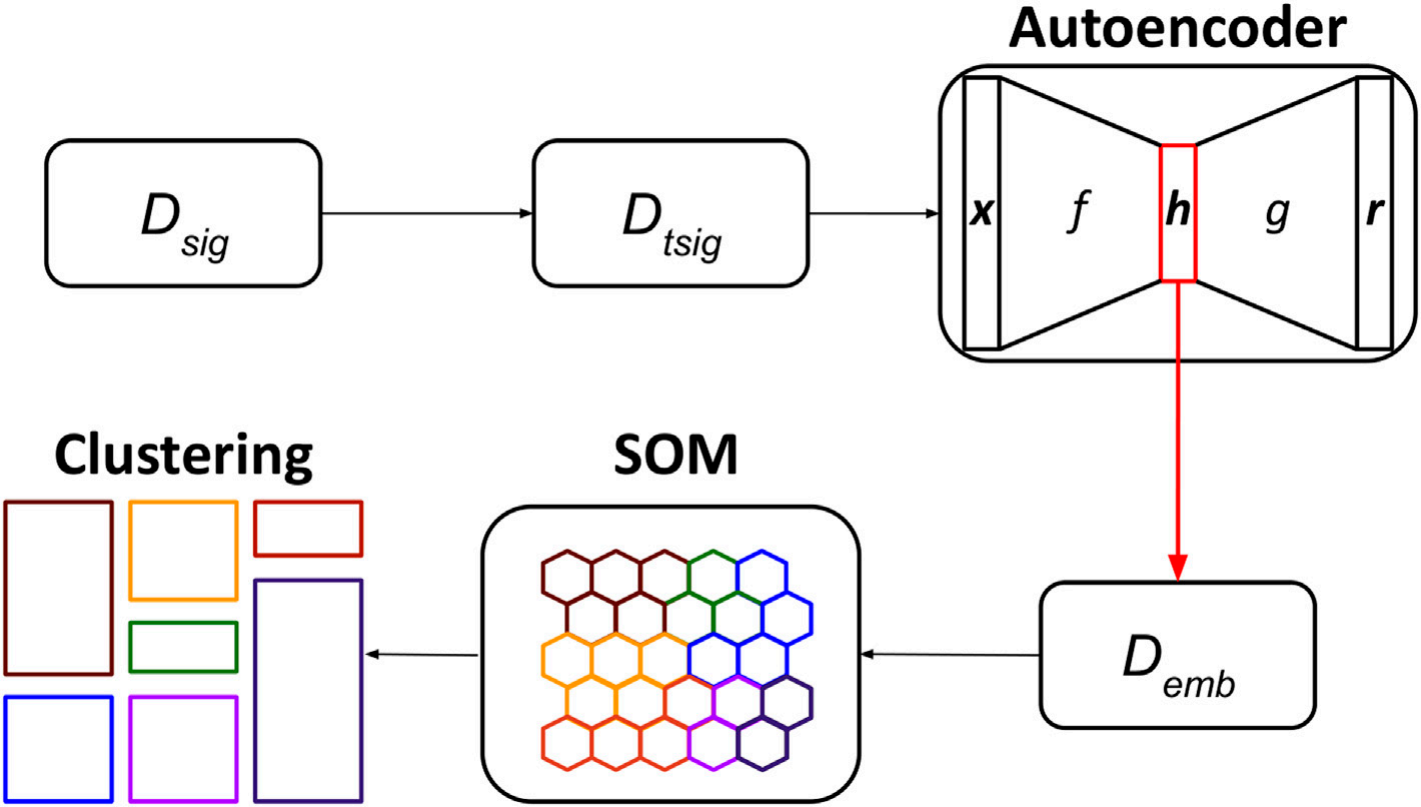
Workflow of the UNPLEX. The dataset containing ONT-like signals (*D*_*seq*_) is pre-processed to achieve a dataset of signals of the same length (*D*_*tsig*_), which are fed to the autoencoder to obtain their latent representations (*D*_*emb*_). The data included in dataset *D*_*emb*_ are processed by the SOM, whose outcome is finally clustered, resulting in the classification of ONT-like signals according to their BCs.

The UNPLEX framework leverages a pipeline composed of three unsupervised machine learning techniques—autoencoder, SOM, clustering—to cluster ONT-like raw signals according to their BCs (Figure 4). We opted for the autoencoders because they can build compact data representations that can be effectively clustered (see, for example (Peng et al., 2018; Lim et al., 2020)); the SOM was instead chosen considering its capability to perform clustering by keeping different representatives for each cluster.

In the first step of the pipeline, the raw signals in the dataset *D*_*sig*_ are pre-processed to generate the dataset *D*_*tsig*_; then, the convolutional autoencoder is trained by exploiting all available signals and produce their latent representations, which are collected in dataset *D*_*emb*_. In the second step, the SOM is trained using all embeddings in *D*_*emb*_, and a BMU is assigned to each embedding. In the third step, the BMUs are clustered using an agglomerative approach to identify the neurons of the SOM that represent the embeddings of all raw signals sharing the same BC (represented in Figure 4 with different colors).

To enhance the performance of UNPLEX, we perform the hyper-parameter optimization by means of SMBO, using a random forest as a surrogate model (Breiman, 2001). Random forests were chosen as they are more suited to handle discrete hyper-parameters as compared to other approaches like, for instance, Gaussian processes. In particular, we optimize the number of neurons of a square SOM, the learning rate, the neighborhood function (including the respective starting value of standard deviation), the network topology and the distance function. The considered values for each parameter are reported in Supplementary Table S4.

UNPLEX is fully developed with Python 3 and leverages three module packages: Keras, to build and train the autoencoder (Gulli and Pal, 2017); minisom, to implement the SOM (Vettigli, 2018); SMAC3, to perform the hyper-parameter optimization step (Lindauer et al., 2022).

## 3 Results

Two *in silico* generated datasets *D*_1_ and *D*_2_ are used to test the capability of the unsupervised machine learning pipeline UNPLEX to demultiplex BCs ONT-like raw signals. *D*_1_ and *D*_2_ consists in 50 000 and 100 000 signals, respectively, derived from a number of *A* = 50 (BCs) and *B* = 1 000 (UMIs) for *D*_1_, and *A* = 100 and *B* = 1 000 for *D*_2_. The chosen value of *A* represents the number of cells in each dataset, and *B* roughly represents the number of transcripts derived from each cell. Given the different size of the two initial datasets, the hyper-parameters of UNPLEX are optimized by leveraging a budget of 60 MEs for *D*_1_, and 30 MEs for *D*_2_ due to the higher computational costs. The score function used to guide the optimization process of SMBO is defined as the average between the FMS and ARS score values. For both datasets, the number of clusters to be identified by UNPLEX is set equal to *A*, i.e., the number of BCs present in the datasets.

**FIGURE 5:**
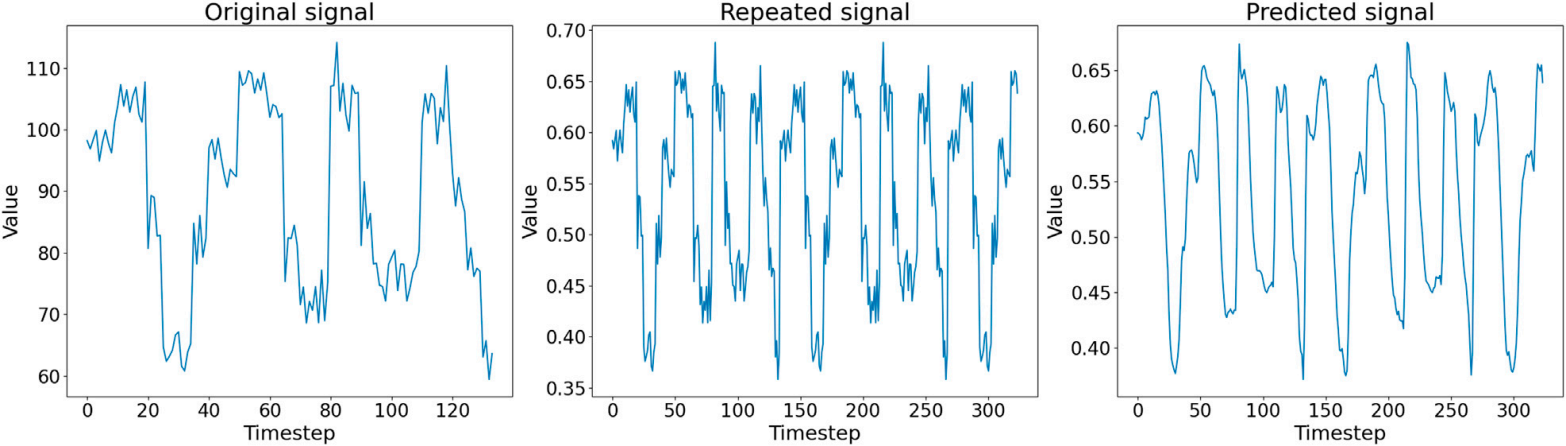
(*Left*) First half of an *in silico* generated signal in *D*_*sig*_. (*Middle*) Pre-processed signal in *D*_*tsig*_ given as input to the autoencoder. (*Right*) Corresponding signal representation generated as output by the autoencoder.

In Figure 5, we show an example of the first half of an *in silico* generated signal (left panel), the corresponding pre-processed signal in *D*_*tsig*_ (middle panel), and the signal reconstructed by the autoencoder (right panel). Noteworthy, the autoencoder output signal is a *de-noised* version of the input signal: the embedding does not contain any information about the noise introduced by the recording process of the nanopore. At the end of the training, the reconstructed signals are thus similar but not identical to the input signals, suggesting that the autoencoder successfully learn an embedding that can be used to reconstruct the input.

**FIGURE 6:**
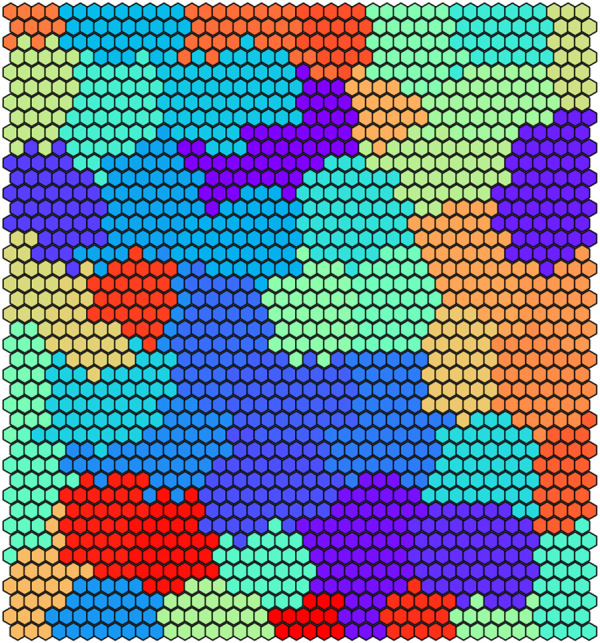
Graphical representation of the 50 clusters identified by the SOM on dataset *D*_1_. Each hexagon represents a neuron colored according to the cluster it belongs to, that is, the BC it represents.

**FIGURE 7:**
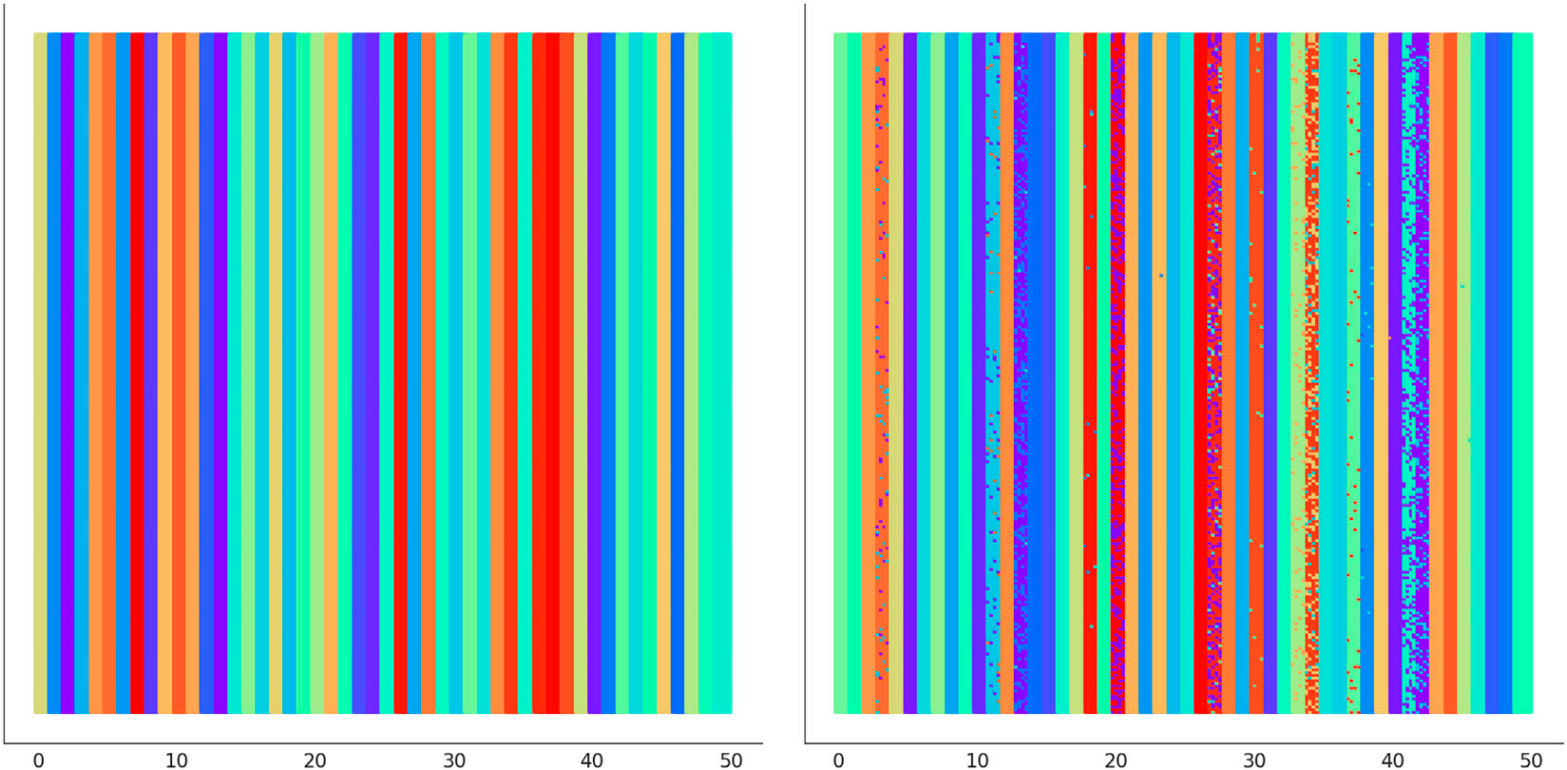
(Left) Gold standard clustering where each bar corresponds to all the signals pertaining the same BC in dataset *D*_1_. (Right) Clustering outcome where the signals are colored according to the result achieved with UNPLEX, non-uniform coloring in a bar indicates the presence of misclustered signals.

In Figure 6 we illustrate the clustering performed by UNPLEX on *D*_1_ with the best hyper-parameterization found by SMBO. Each SOM’s neuron is highlighted with a color representing the cluster it is assigned to. Figure 7 provides a different representation of this result. Specifically, the left plot shows the gold standard clustering, in which all signals in *D*_1_ are perfectly grouped according to their BC, producing a sequence of uniformly colored vertical bars. In the right plot, the signals are displayed with the colors corresponding to the clusters they are assigned to by UNPLEX, and are arranged in vertical bars that can follow a different order with respect to those of the gold standard. If the right panel shows only uniformly colored columns, this means that UNPLEX perfectly clusters all the signals, i.e., both FMS and ARS are maximized. In the case of Figure 7, we observe that some columns are characterized by more than one color. Such phenomenon indicates that some signals are inserted in the wrong cluster by UNPLEX. Non-etheless, the performance metrics for *D*_1_ (ARS = 0.90 and FMS = 0.92), which provide a numerical indication of the clustering results, confirm the good outcome achieved by UNPLEX.

**FIGURE 8:**
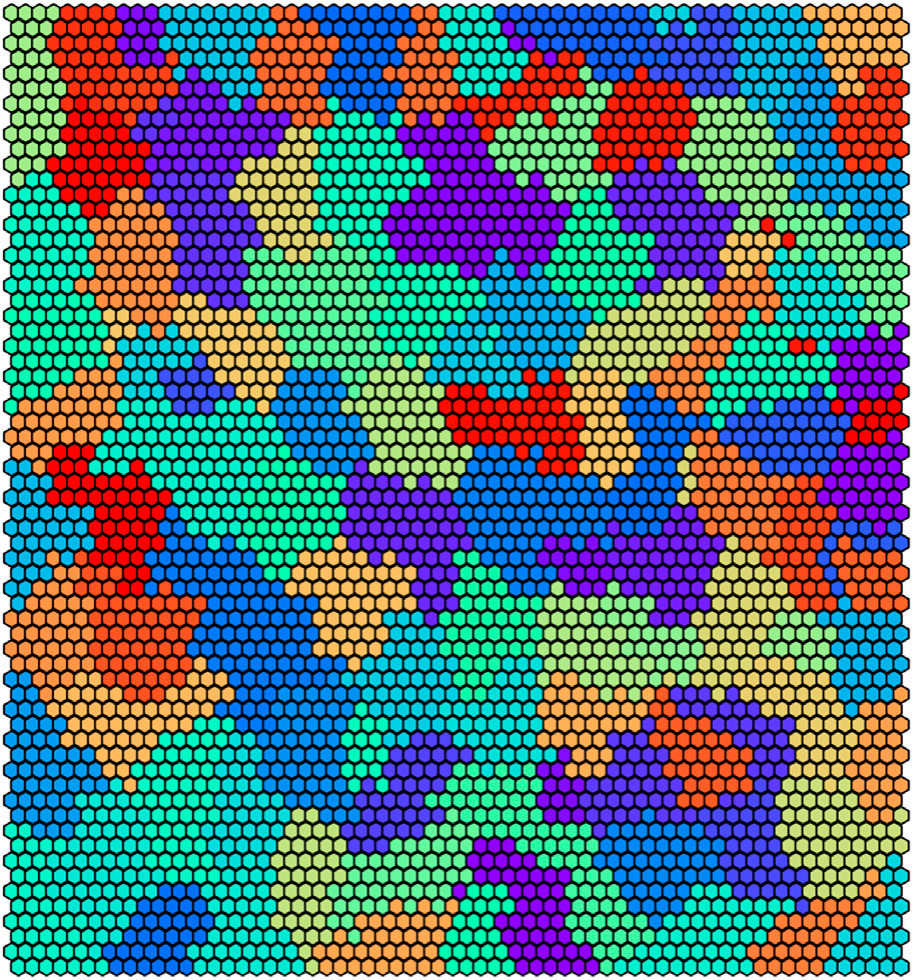
Graphical representation of the 100 clusters identified by the SOM on dataset *D*_2_. Each hexagon represents a neuron colored according to the cluster it belongs to, that is, the BC it represents.

**FIGURE 9:**
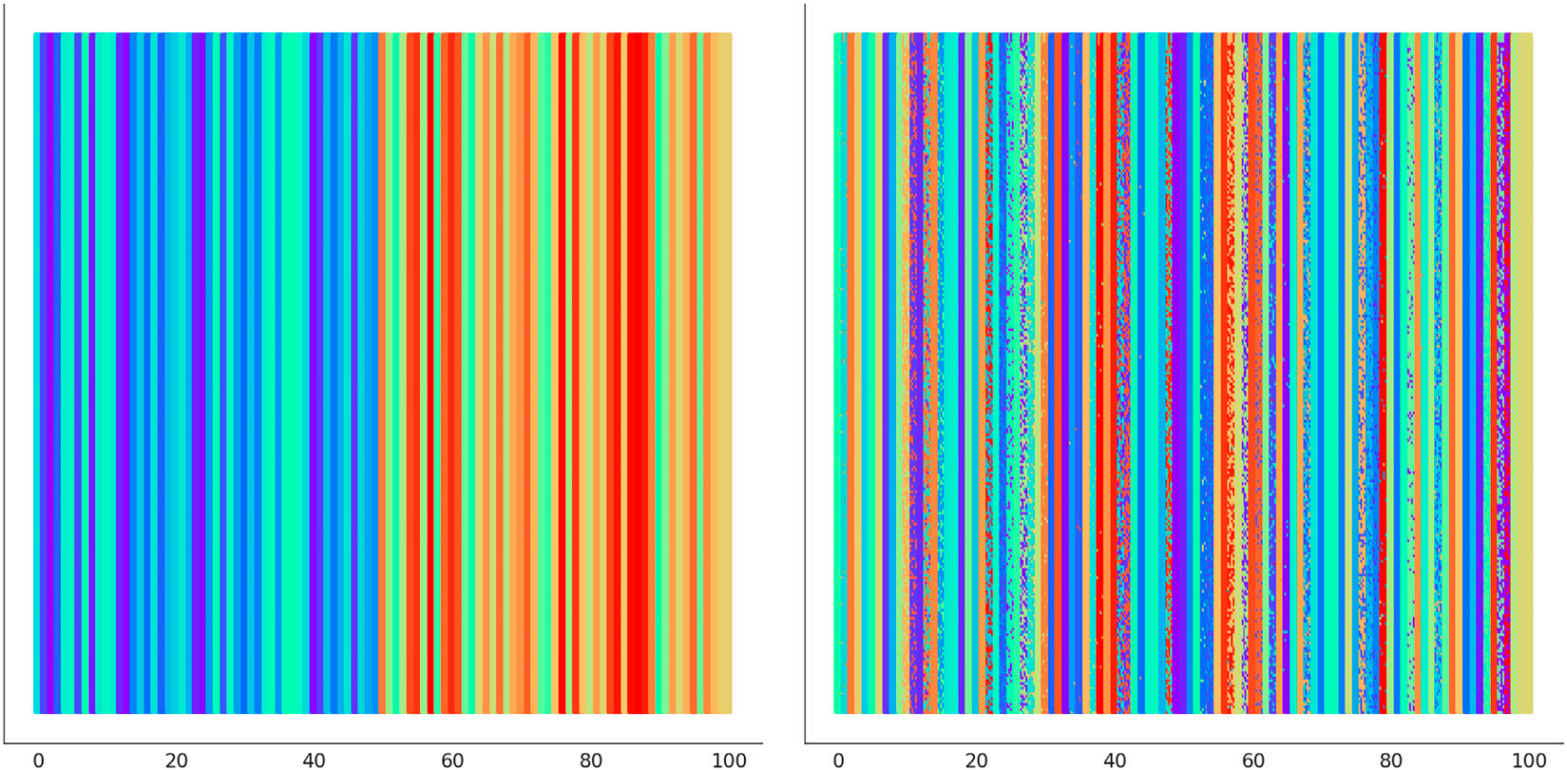
(Left) Gold standard clustering where each bar corresponds to all the signals pertaining the same BC in dataset *D*_2_. (Right) Clustering outcome where the signals are colored according to the result achieved with UNPLEX, non-uniform coloring in a bar indicates the presence of misclustered signals.

Figure 8 reports the clustering obtained by UNPLEX on *D*_2_ with the best parameterization found by SMBO. Figure 9 shows the gold standard (left) and the results obtained by UNPLEX with dataset *D*_2_ (right). In this case, although the column structure is maintained in most cases, the percentage of misclustered signals is increased with respect to the results obtained on dataset *D*_1_. Indeed, the performance metrics indicate a slight decrement of the quality of the clustering (ARS = 0.82 and FMS = 0.81) but yet confirm the effectiveness of UNPLEX.

We argue that the difference in performance between *D*_1_ and *D*_2_ is not caused by the inherent stochasticity of the employed methods, but by the higher number of BCs considered in *D*_2_. Indeed, since the number of BCs in *D*_2_ is twice the amount of *D*_1_, the dissimilarity of the signals produced by different BCs decreases, causing an increase in the error rate of UNPLEX. In fact, in the tests performed on both datasets, we observe that the misclustered signals that should have been assigned to a specific cluster, are instead all inserted into a unique other cluster, i.e., UNPLEX assigns these signals to only one other BC. We hypothesize that this behavior is caused by the fact that such signals are assigned to BMUs of the SOM that lie on the border between two clusters. Stated in other words, considering the graphical representations of the SOM in Figures 6, 8, these BMUs are hexagons adjacent to at least one hexagon having a different color. Specific tests regarding this issue might be helpful to determine which signals are on the borders between two regions, in order to mark them as “unknown” and let the user decide whether to keep them in the current analysis or to consider this subset of signals in a further, more focused experiment to the aim of correctly discriminate the misclustered signals.

## 4 Discussion

Long-read sequencing methods such as ONT, coupled with high-throughput single-cell sequencing platforms, hold the promise to greatly expand the scope of scRNA-seq analyses towards multi-omics approaches, enabling the exploration of additional layers of biological complexity (Kovaka et al., 2023). In this work, we presented UNPLEX, a new computational framework devised to match and demultiplex signals from ONT sequencing with corresponding BC in the coupled scRNA-seq experiment. Specifically, UNPLEX leverages two types of unsupervised neural networks (i.e., a convolutional autoencoder and a SOM), in order to cluster signals containing the same BC.

In our tests, UNPLEX reaches high values of ARS and FMS on two datasets containing 50 000 and 100 000 signals; this result highlights that the raw signals can be properly clustered according to their BC. Contrary to other published methods (Lebrigand et al., 2020; Tian et al., 2021; Ebrahimi et al., 2022), UNPLEX could be successfully applied to demultiplex a relatively small dataset of ONT reads, without the need for expensive high-throughput experiments. Moreover, UNPLEX does not rely on a basecaller to infer nucleotide sequences from Nanopore signals, making its performance independent from the low accuracy of the official ONT basecaller.

The main drawback of the current implementation of UNPLEX is related to the running time, caused by the quadratic scale with respect to the SOM dimension, and the necessity to tune the SOM hyper-parameters to obtain a better clustering of the embeddings. A possible strategy to limit the impact of the computational costs would be analyzing the relationship between the hyper-parameters and the clustering quality of datasets composed of different numbers of signals. In fact, if the optimal hyper-parameterization depends only on the number of signals in the dataset, and not on the signals themselves, it will be possible to perform preemptive hyper-parameter optimizations for different sizes of datasets generated *in silico*. So doing, when a new, real-world dataset has to be clustered, the optimal hyper-parameters for the respective dataset size can be identified by looking at the previous analysis and at the signals properly clustered without any optimization step.

In the future, we plan to improve UNPLEX with the introduction of a highly parallel implementation of the SOM to alleviate the computational burden introduced by the training step. We will also explore the use of implicit metrics (e.g., the silhouette) to assess the clustering quality instead of leveraging a ground truth. In this way, it would be possible to perform the optimization of hyper-parameters also for datasets whose ground truth is unknown. Finally, we will also consider the development of a novel signal generator that leverages the latest signals reported in the literature, since the *k*-mer models used in this work (Nanoporetech, 2017) are no longer supported by Oxford Nanopore Technologies.

At present, UNPLEX was only tested on *in silico* generated signals due to the great challenge of measuring classification accuracy on a real Nanopore sequencing dataset, where the ground truth is unknown. Indeed, the performance of UNPLEX cannot be compared to existing methods because of two reasons. First and foremost, all existing general-purpose methods rely on basecallers, which cannot (by definition) be considered ground truth. As a matter of fact, UNPLEX could (in principle) be more accurate in clustering similar BCs than a basecaller-based approach, but that would be paradoxically counted as an error, affecting the overall accuracy. Second, all existing methods require a high coverage to yield high quality results, which is not the case for UNPLEX that is designed to be effective using a single ONT experiment (i.e., with coverage equal to 1). We are currently working on the generation of a reference dataset to further assess the performance of UNPLEX. Generating ground truth data that include known barcodes typically requires implementing a separate protocol for creating synthetic datasets. A recommended approach would use deep learning techniques to simulate and verify the dataset, taking into account the 5%–15% error rate typically observed in nanopore data due to substitution, deletion, and insertion events. In any case, the obtained signals should be clean enough to avoid misinterpretation by the Dynamic Time Warping (DTW) method, which requires an additional step to pre-process the signals. A validation step of the generated signals is also necessary to make sure they are very close to reality. DTW is a technique used to compare time series data that may have variations in the timing and alignment of their features. Applying DTW to barcode identification in Nanopore sequencing data can be challenging due to the computational complexity of the algorithm, the need for appropriately selecting parameter values, and the requirement for sufficient training data. However, with careful optimization and pre-processing, DTW can be a powerful tool for identifying barcodes in Nanopore sequencing data with high accuracy and enhancing UNPLEX performance.

Although our data generation procedure aimed to faithfully represent a Nanopore signal generated by scRNA-seq libraries, the analysis of real sequencing datasets will likely pose additional challenges in terms of signal complexity and noise profile. Future experimental and analytical work will allow us to extend the applicability of UNPLEX to real Nanopore sequencing datasets. In any case, novel nanopore models and strategies to control the speed of the DNA/RNA strand in the pore are nowadays under investigation by many research groups; therefore, UNPLEX need to be equipped with additional features to keep up with the ONT that is currently evolving (Ying et al., 2022).

Finally, we plan to further improve our methods by studying the neurons of the SOM which fire in response to signals derived from different BCs. In fact, if such neurons are located on the borders between two groups of neurons representing two different BCs, a fine-grained analysis can be performed to mark these signals as “unsure” to the user. Another possible strategy to mitigate this issue is to perform an additional clustering on signals whose BMUs are located on the borders. To this aim, it could be possible to develop a “two-step” framework whereby, after the execution of UNPLEX, only the signals located at the borders are considered and further clustered. During this second step, larger embeddings could be computed to introduce in the compact representations the features that might help to properly cluster such signals; then, such embeddings can be clustered by leveraging a SOM or another clustering approach. Larger embeddings would be computed only for a small subset of signals, since computing larger embeddings for the whole dataset would significantly increase the computational burden, thus making UNPLEX unfeasible due to computational or time limitations. We envision that UNPLEX could be easily adapted to cluster different types of signals, for example, to discriminate UMIs or methylated nucleotide sequences.

## Data Availability

The original contributions presented in the study are included in the article/Supplementary Material, further inquiries can be directed to the corresponding authors.
