# Supplementary material for: Barcode demultiplexing of nanopore sequencing raw signals by unsupervised machine learning
Source: Front Bioinform. 2023 Apr 27;3:1067113. doi: 10.3389/fbinf.2023.1067113 (PMC10173771; doi:10.3389/fbinf.2023.1067113)
Supplement: Supplementary file 1 [file DataSheet1.PDF]

Supplementary Material

Table S1. Topology of the autoencoder used in UNPLEX.

Layer	Activation function
Convolution: kernel = 3, filters = 16	ReLU
Convolution: kernel = 3, filters = 32	ReLU
Max pooling: pool = 2	—
Convolution: kernel = 3, filters = 64	ReLU
Max pooling: pool = 2	—
Max pooling: pool = 2	—
Convolution: kernel = 3, filters = 16	ReLU
Max pooling: pool = 2	—
Up sampling: pool = 2	—
Convolution: kernel = 3, filters = 16	ReLU
Up sampling: pool = 2	—
Convolution: kernel = 3, filters = 64	ReLU
Up sampling: pool = 2	—
Convolution: kernel = 3, filters = 32	ReLU
Up sampling: pool = 2	—
Convolution: kernel = 3, filters = 16	ReLU
Convolution: kernel = 3, filters = 1	Sigmoid

Table S2. Neighborhood functions. The vectors t and u correspond to two neurons of the SOM, the location of t and u on the grid is denoted by their coordinates x and y.

Neighborhood function	Definition
Gaussian	$\exp\left(-\frac{\|t-u\|^2}{2\sigma^2}\right)$
Bubble	$\begin{cases} 1, \text{ if } t_x - \sigma < u_x < t_x + \sigma \wedge t_y - \sigma < u_y < t_y + \sigma \\ 0, \text{ otherwise} \end{cases}$
Mexican hat	$\left(1 - \frac{1}{2}\left(\frac{t^2+u^2}{\sigma^2}\right)\right) e^{-\frac{t^2+u^2}{2\sigma^2}}$
Triangle	$\begin{cases} (\sigma - \|t-u\|), \text{ if } \|t-u\| \leq \sigma \\ 0, \text{ otherwise} \end{cases}$

Table S3. Distance functions. The vector v is the input vector and u is a neuron of the SOM.

Distance function	Definition
Cosine	$\frac{v \cdot u}{\|\|v\|\| \cdot \|\|u\|\|}$
Minkowsky	$\left(\sum_{i=1}^{d} \|\|v_i - u_i\|\|^p\right)^{1/p}$
Manhattan	$\sum_{i=1}^{d} \|\|v_i - u_i\|\|$
Euclidean	$\sqrt{\sum_{i=1}^{d}(v_i - u_i)^2}$
Chebyshev	$\lim_{p\to\infty} \left(\sum_{i=1}^{d} \|\|v_i - u_i\|\|^p\right)^{1/p}$

Table S4. SOM hyper-parameters and corresponding values considered for optimization process.

Hyper-parameter	Values
Number of neurons	$\{35, 36, \ldots, 60\}$
Learning rate	$[0.3, 5.0]$
Neighborhood function	Gaussian, Bubble, Mexican hat, Triangle
Standard deviation for Gaussian and Mexican hat	$[0.5, 3.0]$
Standard deviation for Bubble*	$\{1, 3\}$
Standard deviation for Triangle*	$\{1, 2, 3\}$
Distance function	Cosine, Manhattan, Euclidean, Chebyshev
Topology	Square, Hexagonal

*The intervals considered are discrete since this function leverages only integer values.
